# Supplementary material for: AlphaPept: a modern and open framework for MS-based proteomics
Source: Nat Commun. 2024 Mar 9;15:2168. doi: 10.1038/s41467-024-46485-4 (PMC10924963; doi:10.1038/s41467-024-46485-4)
Supplement: Supplementary file 1 — Supplementary Information [file 41467_2024_46485_MOESM1_ESM.pdf]

Supplementary Material

AlphaPept, a modern and open framework for MS-based proteomics

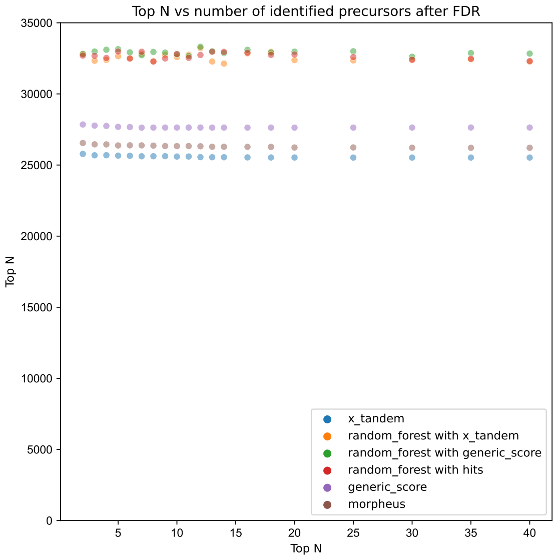

Supplementary Figure 1: Influence of Top-N on the number of identified precursors after FDR. For classical scores such as X!Tandem, Morpheus or our novel generic score, the number of identified precursors slightly decreases with higher N.

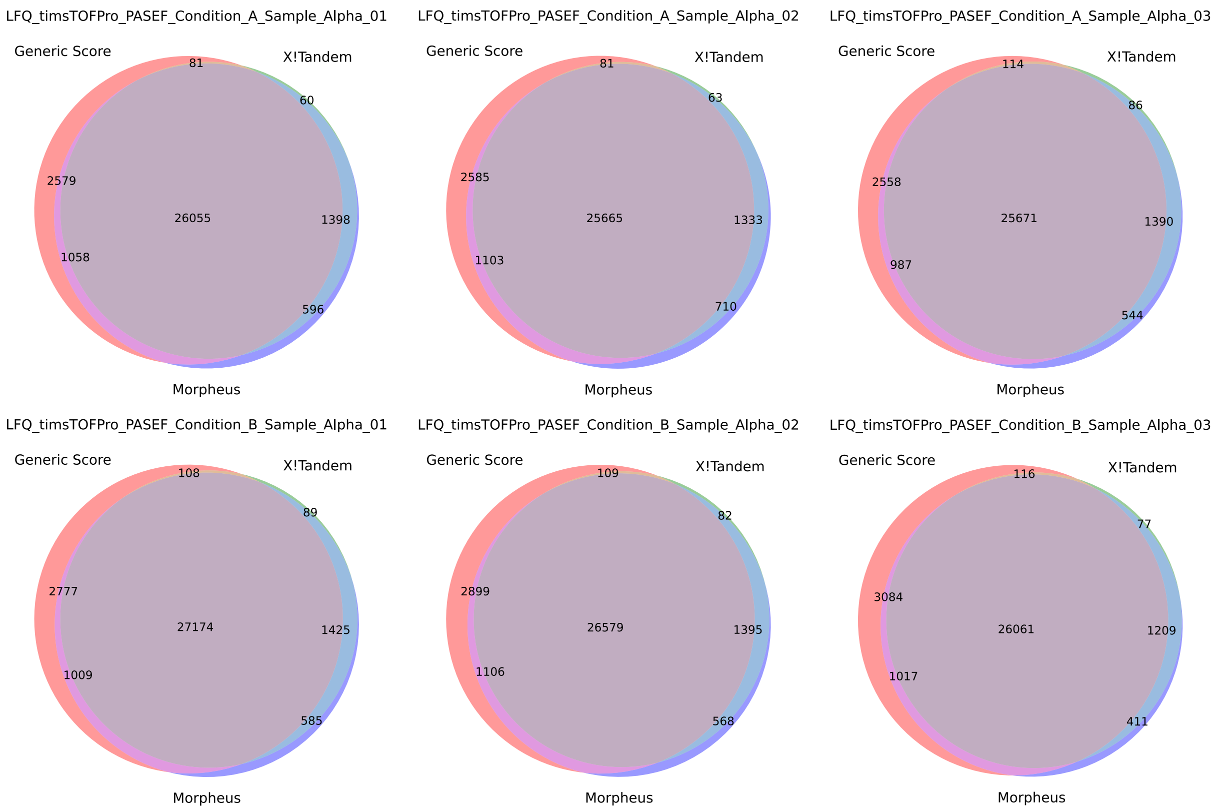

Supplementary Figure 2: Overlap of identifications of the generic score after peptide FDR against X!Tandem and Morpheus on a Thermo dataset from PXD028735 . The overlap is on average, 95% for X!Tandem and 94% for Morpheus. The increase in number of identifications is approx. 5%.

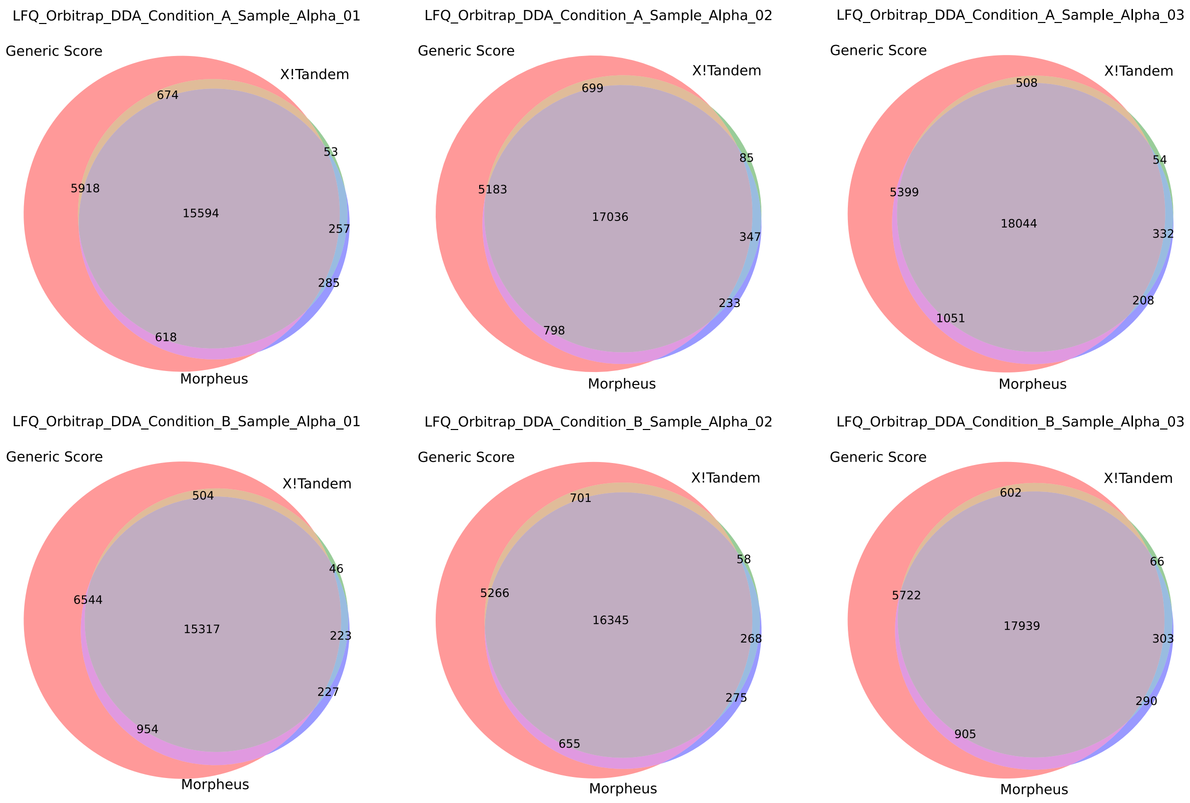

Supplementary Figure 3: Overlap of identifications of the generic score after peptide FDR against X!Tandem and Morpheus on a Thermo dataset from PXD028735 . The overlap is on average, 98% for X!Tandem and 97% for Morpheus. The increase in number of identifications is approx. 33%.

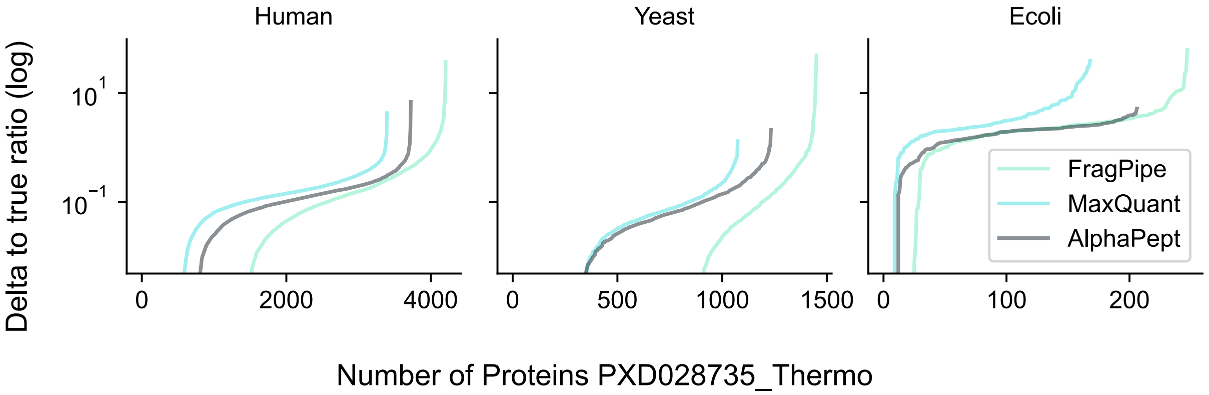

Supplementary Figure 4: Deviation of identifications for mixed-species dataset over the number of identified proteins. The x axis shows the number of proteins, y axis the delta to the true ratio.

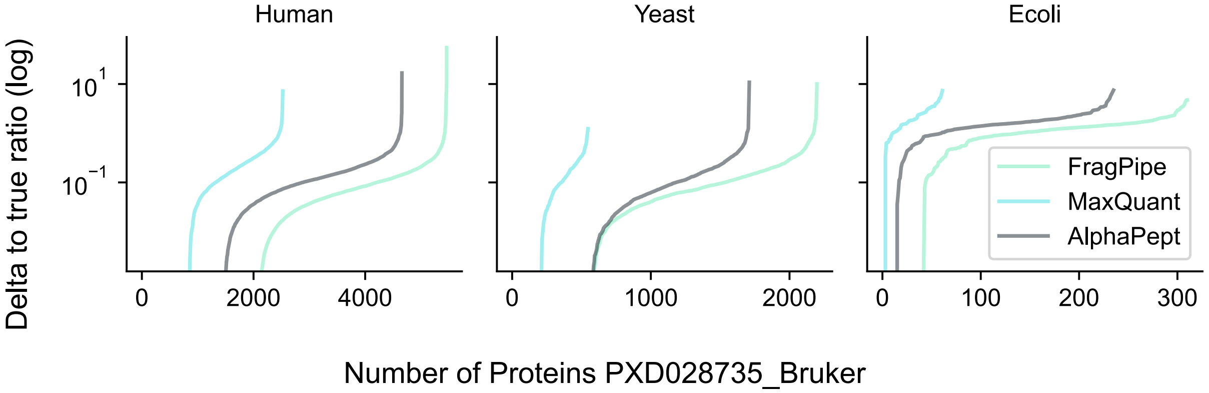

Supplementary Figure 5: Deviation of identifications for mixed-species dataset over the number of identified proteins. The x axis shows the number of proteins, y axis the delta to the true ratio.

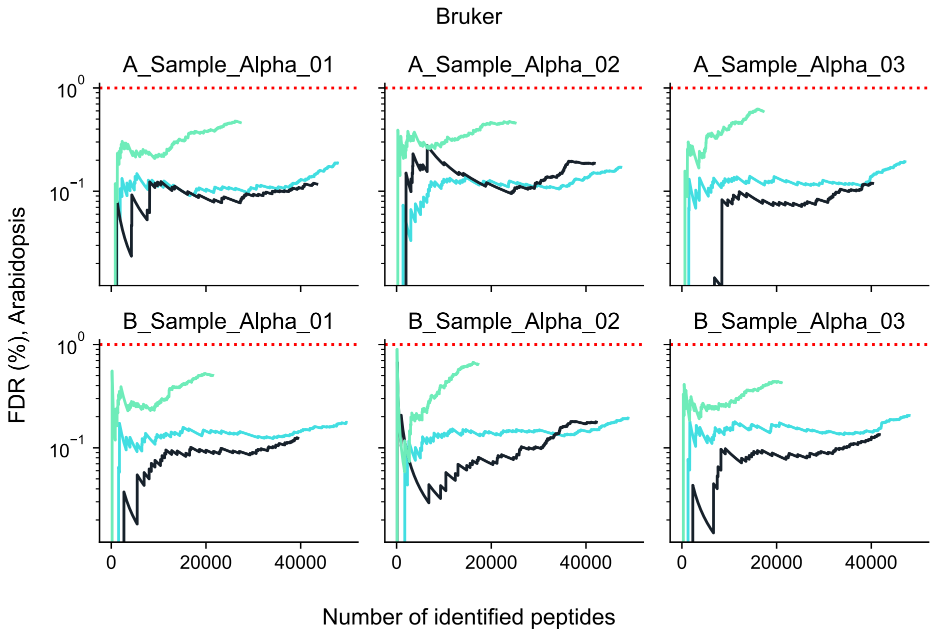

Supplementary Figure 6: Number of identified peptides vs. percentage of Arabidopsis hits for the Bruker dataset from PXD028735 compared across FragPipe (teal), MaxQuant (green) and AlphaPept (black).

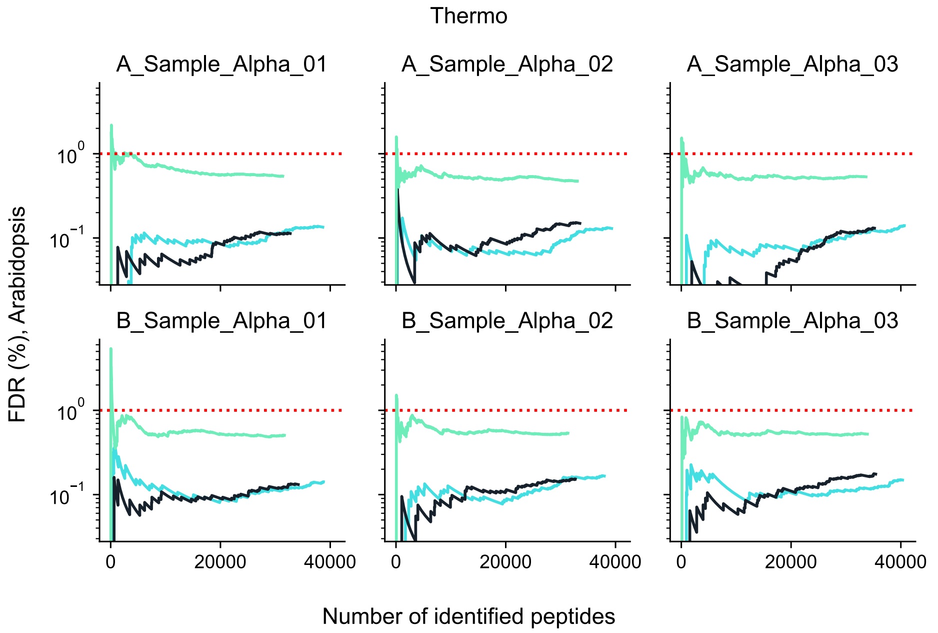

Supplementary Figure 7: Number of identified peptides vs. percentage of Arabidopsis hits for the Thermo dataset from PXD028735 compared across FragPipe (teal), MaxQuant (green) and AlphaPept (black).

$$hits \cdot \frac{hits}{2 \cdot n_{frags\ db}} + hits \cdot fragments\ matched\ int\ ratio + hits_y$$

Supplementary Formula 1: Formula to calculate our generic score.

200 HeLa Proteomes	**Cloud I** AWS (c6i.32xlarge) Windows Server 3.5 GHz x 128 192 GB RAM	**Cloud II** AWS (m5.metal) Windows Server 3.1 GHz x 96 384 GB RAM	**SLURM Cluster I*** Linux 2.1 GHz x 24 384 GB RAM	**SLURM Cluster II**** Linux 2.1 GHz x 24 384 GB RAM	**Reference: Local** Windows 10 3.5 GHz x 24 128 GB RAM
Total Processing Time	444	482	1948	280	413
Per File	2.22	2.41	9.74	1.4	2.1

Supplementary Table 1: Running times of AlphaPept (v.0.5.0) for cloud and Cluster (timings in minutes)
* SLURM Cluster is Linux based. For Thermo files, AlphaPept needs Mono and currently does not allow multiprocessing, so only one file at a time was processed/converted.
** using preprocessed files and multiprocessing

		**Laptop** Macbook Pro macOS Big Sur i9 2.3 GHz x 8 32 Gb RAM	**Office Pc** Optiplex 7080 Windows 10 i9 3.7 GHz x10 64 Gb RAM	**Workstation** Custom Windows 10 i9 3.5 GHz x12 128 Gb RAM	**Cloud I** AWS (t3a.2xlarge) Windows Server EPIC 2.2 GHz x4 32 Gb RAM	**Cloud II** AWS (t3.xlarge) Windows Server XEON 2.4 GHz x2 16 Gb RAM
IRT Sample* (Thermo)	Full	1	1	2	3	2
HeLa 120 min (Thermo)	Full	23	16	19	40	41
	Preprocessed	6	4	5	11	12
PXD006109–- 6 files (Thermo)	Full	36	17	21	46	73
	Preprocessed	30	8	s	18	24
IRT Sample (Bruker)	Full	**	1	2	3	2
HeLa 120 min (Bruker)	Full	**	57	111	131	399
	Preprocessed	6	6	7	16	19
PXD010012–- 10 files (Bruker)	Full	**	242	194	790	893
	Preprocessed	62	24	23	85	132

Supplementary Table 2: Running times of AlphaPept (v0.3.26) for various hardware (timings in minutes)
* IRT = low complexity mixture of peptides (internal retention time standard)
** to process Bruker files on Mac Os X, we preprocessed them on Windows
AlphaPept can be readily employed with cloud providers such as Amazon Web Services. We tested our default testing pipeline (see timing table below) on two different Amazon EC2 instances (t3a.2xlarge: 0.42 Eur/h and t3.xlarge: 0.22 Eur/h), an incurred computational costs of 0.22 and 3.82 Euros for one 120 min Orbitrap HeLa file and 8 timsTOF files, respectively, when processed in a European location. Computational costs can be further improved by choosing resource-optimized hardware or buying computing power in advance.
